# Influence of plant genotype and soil on the cotton rhizosphere microbiome

**DOI:** 10.3389/fmicb.2022.1021064

**Published:** 2022-09-20

**Authors:** Chuanzhen Yang, Hongchen Yue, Zheng Ma, Zili Feng, Hongjie Feng, Lihong Zhao, Yalin Zhang, Greg Deakin, Xiangming Xu, Heqin Zhu, Feng Wei

**Affiliations:** ^1^State Key Laboratory of Cotton Biology, Institute of Cotton Research of Chinese Academy of Agricultural Sciences, Anyang, China; ^2^School of Agricultural Sciences, Zhengzhou University, Zhengzhou, China; ^3^NIAB East Malling Research, Kent, United Kingdom

**Keywords:** *Gossypium barbadense*, *Gossypium hirsutum*, *Verticillium dahliae*, rhizosphere soil, amplicon-sequencing, microbial community composition

## Abstract

Rhizosphere microbial communities are recognized as crucial products of intimate interactions between plant and soil, playing important roles in plant growth and health. Enhancing the understanding of this process is a promising way to promote the next green revolution by applying the multifunctional benefits coming with rhizosphere microbiomes. In this study, we propagated eight cotton genotypes (four upland cotton cultivars and four sea-land cotton cultivars) with varying levels of resistance to *Verticillium dahliae* in three distinct soil types. Amplicon sequencing was applied to profile both bacterial and fungal communities in the rhizosphere of cotton. The results revealed that soil origin was the primary factor causing divergence in rhizosphere microbial community, with plant genotype playing a secondary role. The Shannon and Simpson indices revealed no significant differences in the rhizosphere microbial communities of *Gossypium barbadense* and *G. hirsutum*. Soil origin accounted for 34.0 and 59.05% of the total variability in the PCA of the rhizosphere bacterial and fungal communities, respectively, while plant genotypes within species only accounted for 1.1 to 6.6% of the total variability among microbial population. Similar results were observed in the Bray–Curtis indices. Interestingly, the relative abundance of Acidobacteria phylum in *G. barbadense* was greater in comparison with that of *G. hirsutum.* These findings suggested that soil origin and cotton genotype modulated microbiome assembly with soil predominantly shaping rhizosphere microbiome assembly, while host genotype slightly tuned this recruitment process by changing the abundance of specific microbial consortia.

## Introduction

Rhizosphere microbiome can play a critical role in improving plant health and productivity (Töwe et al., 2010; Mohite, 2013; Parmar and Sindhu, 2013; Haney et al., 2015; Kramer et al., 2020; Wei et al., 2021). Recent research on the composition and function of soil microbial community, particularly rhizosphere, has provided insights into how they may promote plant growth and improve disease resistance. Several variables, including host genotype and soil pH, may form the rhizosphere microbial population (Peiffer et al., 2013; Schlaeppi et al., 2014; Zhong et al., 2019; Han et al., 2020; Trivedi et al., 2020). However, elucidating the mechanism by which rhizosphere microbiota interacts with plants to enhance plant growth remains difficult.

Soil, as the seed bank of rhizosphere microorganisms, is recognized as a crucial factor that influences the assembly of rhizosphere microbial community (Veach et al., 2019). Soil physicochemical properties (e.g., pH, nutrients, and texture) can considerably affect rhizosphere microbial community directly or indirectly through regulating plant root exudates (Lundberg et al., 2012; Fitzpatrick et al., 2020). Compared with genotypes and chemical types, soil origin can explain 47 and 33% of the variation of underground bacterial and fungal communities, respectively, and is the main habitat filter leading to differences in underground microbial communities, followed by genotypes (Veach et al., 2019). Understanding the relative importance of those factors affecting rhizosphere microbiome is a prerequisite for targeted manipulation of rhizosphere microbiome in a manner that will boost crop yield (Zhang et al., 2017; Trivedi et al., 2020).

Plant genotype is another important factor affecting the assembly of rhizosphere microorganisms. A large-scale study of rhizosphere microorganisms of 27 maize genotypes showed a close association of many microbial taxa groups with host genotypes, which was also reported in *Arabidopsis* and soybean (Schlaeppi et al., 2014; Walters et al., 2018; Zhong et al., 2019). Recent studies have revealed that plant genotypes that exhibit differential resistance to a specific pathogen may lead to increased abundance of specific microbial consortia, which in turn may alter host performance. For example, *Flavobacterium* was selectively enriched by tomato with resistance to *Ralstonia* when compared with that susceptible to *Ralstonia*, which was further confirmed to participating in mitigating Ralstonia wilt (Kwak et al., 2018).

Verticillium wilt, caused by *Verticillium dahliae*, is a damaging soil-borne disease that affects numerous plant species across the globe. Effective management of Verticillium wilt is difficult to achieve owing to the inaccessibility of fungal infection structure, long-term survival of its microsclerotia in soil, and withdrawal of broad-spectrum soil fumigants (Klosterman et al., 2009). As the most important fiber crop, the allotetraploid cotton (*Gossypium hirsutum* and *G. barbadense*), originating from transoceanic hybridization of an A-genome-like ancestral African species (*G. herbaceum* or *G. arboretum*) with a native D-genome-like species (*G. raimondii*) accounts for about 90% of the global cotton output because of its high fiber yield and wide adaptability (Endrizzi et al., 1985; Wendel, 1989; Zhang et al., 2015). Compared with *G. hirsutum*, *G. barbadense* has superior fiber quality and more importantly is reported to be almost immune to *V*. *dahliae* (Ma et al., 2021). Breeders have been trying to integrate key traits from *G. hirsutum* and *G. barbadense* for many years and have yet to achieve this goal (Ma et al., 2021). Thus, wilt management in cotton (*G. hirsutum*) production remains a key challenge. Our previous study indicated that both rhizosphere and endosphere microbial communities differ between wilt-susceptible and wilt-resistant *G. hirsutum* cultivars (Wei et al., 2019). Rhizosphere of resistant varieties is enriched in Firmicutes, Actinobacteria, and *Trichoderma spp.*

In this study, we report results on the rhizosphere microbiome in relation to cotton species (*G. hirsutum* and *G. barbadense*), cultivars within each species and sites.

## Materials and methods

### Field experiment design

A field experiment was conducted at the Institute of Cotton Research of Chinese Academy of Sciences (Anyang, China) (36°03′44′′N, 114°28′52′′E) to assess Verticillium wilt resistance. A completely randomized block design with three blocks was used with an initial inoculum of 15.2 CFU/g soil based on wet sieving and plating of soil samples on a semi-selective medium. Eight cotton cultivars were included: four upland cotton [*G. hirsutum*] cultivars, namely, cv. Lumianyan21, cv. TM-1, cv. Zhongmiansuo24, and cv. Zhongmiansuo35, and four sea-land cotton [*G. barbadense*] cultivars, namely, *G. barbadense* cv. 3-79, cv. Hai7124, cv. Xinhai21, and cv. Xinhai25. Each block consisted of eight plots which were 5.0 m long with two rows (0.8 m between two rows); the neighboring plots were separated by 1.0 m. In April 2019, seeds were sown with a within-row plant-to-plant distance of 25 cm. During late August, approximately 16 weeks after sowing, wilt severity on all individual plants was recorded on a scale of 0 to 4, and the disease index was calculated as described previously (Wei et al., 2019).

### Soil collection for greenhouse experiment

Soil from a cotton field in Alaer, Xinjiang (40°41′01′′N, 80°41′56′′E), was collected on May 15, 2019, with shovels to a depth of approximately 20 cm, in Shihezi, Xinjiang (44°20′24′′N, 86°01′06′′E), on May 19, 2019, and in Anyang, Henan (36°03′44′′N, 114°28′52′′E), on May 19, 2019. The initial inoculum of microsclerotia of *V. dahliae* for Alaer, Shihezi, and Anyang field soils was 6.4, 4.7, and 15.2 CFU/g dry soils, respectively. The field in Anyang was artificially inoculated with microsclerotia of *V*. *dahliae* in 2000. Cotton (*G. hirsutum*) was grown at the three fields for many years, and Verticillium wilt had been occurring with the severity varying among years. Soils from all three sites were transported back to the greenhouse in the Institute of Cotton Research, Anyang, and stored until sowing on June 21, 2019. Soils from each field were mixed in clean tubs in order to homogenize the soil, before being placed into pots (30 cm in diameter and 20 cm in height). The physicochemical properties of the soils are listed in Table 1.

**TABLE 1:** The physicochemical properties of different site soils.

Site	TN* (g/kg)	Alkaline nitrogen (mg/kg)	Avail-P* (mg/kg)	Avail-K* (mg/kg)	SOM* (g/kg)	pH
Anyang	0.77	64	30.0	184	11.7	7.39
Shihezi	1.52	104	47.7	81	19.7	7.14
Alaer	0.54	31	59.9	223	8.9	7.15

*TN, total carbon; Avail-P, available phosphorus; Avail-K, available potassium; SOM, soil organic matter.

### Experimental design

There were 24 treatments: eight cotton cultivars [four upland cotton (*G. hirsutum*) cultivars, namely, cv. Lumianyan21, cv. TM-1, cv. Zhongmiansuo24, and cv. Zhongmiansuo35, and four sea-land cotton (*G. barbadense*) cultivars, namely, *G. barbadense* cv. 3-79, cv. Hai7124, cv. Xinhai21, and cv. Xinhai25], each grown in three soil types (collected from the three sites). A randomized block design, with three blocks, was used. Within each block, there was a single pot (20 cm diameter, 15 cm high) for each of the 24 treatments. Each pot was filled with 3 kg of soil from one of the three sites, and then, eight seeds of a single cultivar were sown. The pots were placed in a greenhouse with a 12-h/12-h light/dark cycle at 25–28°C and watered regularly. Six days later, seedlings were thinned to five seedlings per pot.

### Sampling rhizosphere soils

Eight weeks after sowing, plants and soils were removed from each pot and the roots were removed from the soil. Any roots that were in contact with the pot were not sampled. The root system was firstly separated from the bulk soil by gently shaking and then shaking more vigorously; the remaining 1–2-mm soil layer adhered to the roots was defined as rhizosphere soil. For each pot, rhizosphere soils from the three plants were pooled together and sieved (2 mm) to form one sample. In total, 72 samples were obtained from rhizosphere soil (3 sites × 2 cotton species × 4 cultivars × 3 replicates). All the soil samples were stored at −80°C before DNA isolation.

### Deoxyribonucleic acid extraction and sequencing protocols

Total DNA from all the 72 samples was extracted using the MoBio PowerSoil DNA Isolation Kit (MoBio Laboratories, Carlsbad, CA, USA). A rhizosphere sample (250 mg) was resuspended in 500 μL of bead solution, and DNA was extracted according to the manufacturer’s instructions. The extraction was examined on 1% agarose gel, and the DNA concentration was estimated with a NanoDrop ND-2000 spectrophotometer (NanoDrop Technologies, Wilmington, DE, USA). The barcoded primers 341F/805R (Herlemann et al., 2011) were used to amplify the V3–V4 hypervariable region of bacterial 16S rRNA, and the primers ITS5/ITS2 (White et al., 1990) were used to amplify the ITS1 region of fungi. PCRs and the extraction and purification of amplicons were performed according to a previously published protocol (Wei et al., 2019). Sequencing libraries were generated with the TruSeq^®^ DNA PCR-Free Sample Preparation Kit (Illumina, San Diego, CA, USA) following the manufacturer’s recommendations. The quality of each library was assessed on a Qubit 2.0 Fluorometer (Life Technologies, USA). Finally, samples were sent to Novogene Bioinformatics Technology Co., Ltd., Beijing, China, for paired-end sequencing on the IonS5™XL platform (Thermo Fisher Scientific, Waltham, MA, USA).

### Sequence processing and analysis

Sequences were processed and filtered separately for 16S and ITS data to retain high-quality sequences. The raw reads were first quality-filtered by Cutadapt (v1.9.1^1^), which were then compared with the reference database^2^ and analyzed by UCHIME algorithm^3^ for detection and removal of chimera sequences. Then, all unique sequence reads were sorted by their respective frequencies and then clustered into operational taxonomic units (OTUs) based on a 97% similarity threshold using the UPARSE pipeline (v10.0), and at the same time, a representative sequence for each OTU was generated (Edgar, 2013). The SINTAX algorithm^4^ was then used to assign each OTU to a taxonomic rank by alignment of the gene sequences against the Unite V7 fungal ITS database (Kõljalg et al., 2013) and the RDP training set (v16) bacterial 16S database (Love et al., 2014) based on a confidence threshold value of 80%. Then, an OTU counts table (a sample-by-observation contingency table) was generated by aligning all sequences (filtered with far less stringent criteria) with the OTU representative sequences at the 97% similarity as described previously (Deakin et al., 2018).

### Statistical data analysis

All sequence summaries per sample and at a specific taxon were calculated directly from the original reads number, whereas all subsequent statistical analyses (alpha and beta diversity analysis, PCA, and DESeq2) were based on the counts normalized with the median of ratios as implemented in DESeq2.

General statistical methodology was similar to that of previous publications (Wei et al., 2019). Alpha diversity indices, including Shannon, Simpson, and observed, were analyzed using the “vegan” 2.3-1 in R statistical software (Dixon, 2003). The results were visualized using the “ggplot2” package. The ranks of alpha diversity indices of different samples were analyzed using permutation based on analysis of variance to evaluate the difference among three soil types. Beta diversity indices were calculated and subjected to non-dimensional scaling analysis as implemented in the “vegan” package. The effects of cultivars, soil types, and two cotton species (*G. hirsutum and G. barbadense*) on the first four principal components were determined *via* ANOVA. Similarly, the effects of these experimental factors on the beta diversity (Bray–Curtis indices) were assessed and subjected to permutational multivariate analysis of variance (PERMANOVA) with 999 permutations.

Further analysis was carried out to identify specific microbial OTUs that differed significantly in their relative abundances between the two cotton species through DESeq2 (McMurdie and Holmes, 2013). DESeq2 also implements an algorithm for automatic filtering of OTUs before differential abundance analysis using several criteria, including variance in abundance across samples and overall abundance level. The Benjamini–Hochberg (BH) adjustment was used with DESeq2 (Benjamin and Aikman, 1995) to correct for the false discovery rate associated with multiple testing.

## Results

### Field disease development

An average wilt index for *G. hirsutum* ranged from 28.05 (cv. Lumianyan21) to 49.07 (cv. TM-1), while that for *G. barbadense* ranged from 0.79 (cv. Hai7124) to 4.40 (cv. 3-79) (Supplementary Table 1). The four *G. barbadense* cultivars were immune to *V. dahliae.*

### Overall sequencing results

For fungal data, the number of raw reads ranged from 67,883 to 111,644 per sample, with an average of 83,230; the number of good quality reads ranged from 48,455 to 105,182, with an average of 79,503. There were 5,768 fungal OTUs; the number of sequences classified into OTUs ranged from 10,727 to 102,169 per sample, with an average of 69,226. There was only one sample that had a very low number of reads, but even for this sample, the number of reads classified into OTUs is still 10,727. The sample with the second lowest number of reads classified into OTUs had 46,258 sequences. Sequencing depth was sufficient for all samples (Supplementary Figure 1A).

**FIGURE 1:**
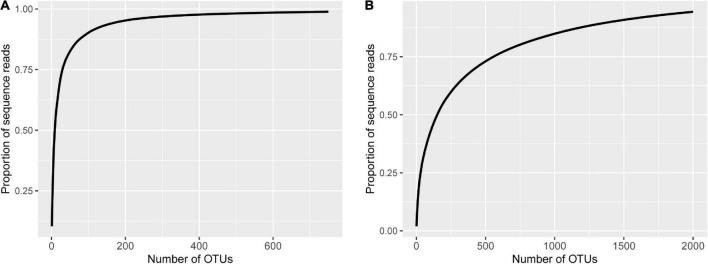
Fungal **(A)** and bacterial **(B)** cumulative proportion of sequence reads plotted against the number of operational taxonomic units (OTUs) where the OTUs were sorted in the descending order of their reads.

The majority of fungal reads were less than 100 OTUs. The most prevalent OTUs accounted for 10.4% of all sequences, with the top 9 and 98 OTUs accounting for more than 50 and 90% of the total number of sequences, respectively (Figure 1A). About half of the sequences (47.0%) cannot be assigned to the phylum level at the 80% confidence; Ascomycota and Zygomycota accounted for 47.4 and 3.2% of sequences, respectively (Supplementary Figure 2A). Most of the Ascomycota sequences were of Sordariomycetes.

For *G. barbadense*, sequences from Ascomycota and un-identified fungal groups accounted for ca. 49.1 and 47.9% of the total sequences, respectively; the corresponding values for *G. hirsutum* were 45.9 and 46.3%. *G. hirsutum* appeared to have more sequence reads of Basidiomycota (3.5%) and Zygomycota (4.0%) than those of *G. barbadense* (0.6 and 2.2%). There were considerable differences in the percentage of sequences in each phylum among the eight cultivars studied, mainly manifested in the proportions of the Ascomycota and “Unknown” groups (Supplementary Figure 3). Similarly, differences among the three soil types were also mainly related to the differences between the Ascomycota and “Unknown” groups.

For bacterial data, the number of raw reads ranged from 78,440 to 99,582 per sample, with an average of 90,407; the number of good quality reads ranged from 57,321 to 80,889, with an average of 72,443. There were 4,941 bacterial OTUs. The number of sequences classified into OTUs ranged from 52,430 to 76,740 per sample, with an average of 65,749. Sequencing depth is sufficient for all samples (Supplementary Figure 1B). Compared with the fungal data, the sequences were more spread among the OTUs (Figure 1). For example, the most prevalent bacterial OTU only accounted for 2.0% of the total number of sequences; the top 151 and 1,402 bacterial OTUs accounted for more than 50 and 90% of the total number of sequences, respectively (Figure 1B). Overall, Proteobacteria accounted for 45.6% of the total reads, followed by the “Unknown” (16.6%) and Actinobacteria (14.6%) (Supplementary Figure 2B). Frequencies of the other phyla ranged from 5 to 16%. Most of the Proteobacteria sequences were of Alphaproteobacteria.

*Gossypium barbadense* and *G. hirsutum* differed little in the percentage of sequence reads in individual phyla. However, there were noticeable differences in the frequencies of sequences in individual phyla among the three soil types (Supplementary Figure 4). The soil from Anyang had a higher proportion of Proteobacteria than that from the other two sites in the expense of primarily the “Unknown” group.

### Alpha diversity

**FIGURE 2:**
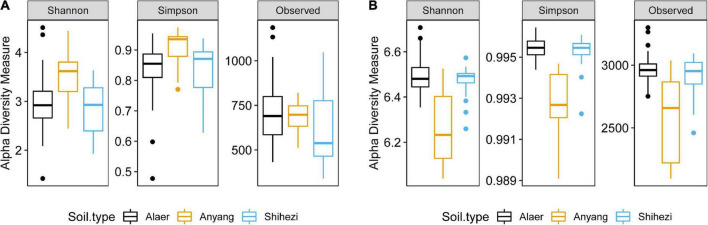
Fungal **(A)** and bacterial **(B)** alpha diversity indices for three soil types.

The three soil types varied (*P* < 0.01) for both the Shannon and Simpson indices, whereas soil grown with *G. barbadense* and *G. hirsutum* did not vary significantly in the two indices. The Simpson and Shannon indices were greater in Anyang soil than those in the other two locations (Figure 2A). Similarly, only the three soil types varied (*P* < 0.001) for both the Shannon and Simpson indices for bacteria. In contrast to fungi, the soil from Anyang had significantly lower Shannon and Simpson indices than that from the other two locations (Figure 2B).

### PCA

**FIGURE 3:**
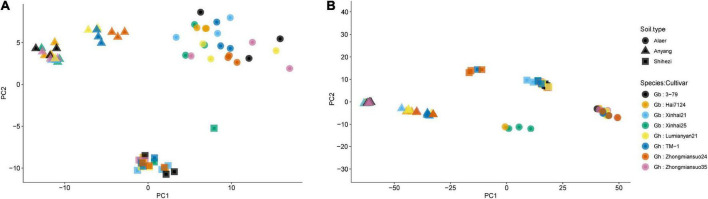
Plot of the first two principal components (PCs) of the fungal communities **(A)** and bacterial communities **(B)** on the rhizosphere of eight cotton cultivars grown in the soils collected from the three sites: four cultivars from *Gossypium barbadense* (GB) and the other four from *Gossypium hirsutum* (GH).

For fungi, the first two PCs accounted for 19.6 and 17.5% of total variance in the observed data, respectively, whereas the third and fourth PCs only accounted for 6.3 and 3.1%, respectively. The most variability in the first two PCs was due to the differences among the three soil types, accounting for 86.5 and 96.5% of the total variability, respectively (Figure 3A and Table 2). Samples from plants grown in the soil from Alaer appeared to be more variable than other samples (Figure 3A). In addition, samples from plants grown in the soil from Anyang seemed to be divided into two groups, but not all related to cultivars (Figure 3A). Differences between the two species or cultivars within each species accounted for a small proportion of the total variability, though occasionally statistically significant (Table 2). Overall, the soil type explained about 34.0% of the total variability in the data, whereas species and cultivars within species only accounted for the respective 1.4 and 6.6% of the total variability.

**TABLE 2:** Summary of ANOVA of both fungal and bacterial PCAs as a percentage of variability in the first four PCs accounted for by experimental factors (soil types, comparison between two cotton species, cultivars within each species, and residual).

Terms	Fungi					Bacteria				
		
	PC1	PC2	PC3	PC4	Overall	PC1	PC2	PC3	PC4	Overall
Soil types	86.5***	96.5***	3.1	0.1	34.0	88.4***	87.6***	10.0*	4.5	59.0
Gb vs. Gh	1.1*	0.1	3.6	0.6	1.4	0.7	0.1	0.1	5.6	1.1
Species/cultivars	1.1	0.3	17.8*	7.9	6.6	1.6	1.0	6.4	19.1*	4.0
Residual	11.3	4.1	75.6	91.4	58.0	9.3	11.3	83.4	70.9	35.9
Variance due to PC	19.6	17.5	6.3	3.1		50.3	16.2	2.2	1.8	

* and *** indicate the statistical significance at the level of 5 and 0.1%, respectively.

The overall pattern for the bacterial community is similar to the one observed for the fungal community. The first two PCs accounted for 50.3 and 16.2% of the total variability, respectively (Table 2). The most variability in the first two PCs was due to the differences among the three soil types, accounting for the respective 88.4 and 87.6% of the total variability (Figure 3B and Table 2). In contrast to fungi, samples appeared to be divided into two distinct groups irrespective of soil origins, but not all related to cultivars. Overall, the soil type explained nearly 59.0% of the total variability, whereas species and cultivars within species only accounted for the respective 1.1 and 4.0% of the total variability.

### Beta diversity: Bray–Curtis indices

**FIGURE 4:**
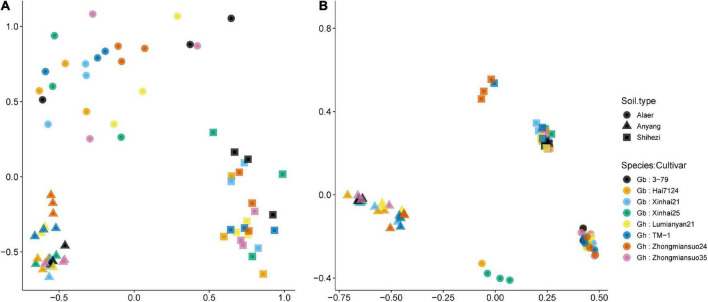
Plot of the first two dimensions of the NDMS analysis for the fungal **(A)** and bacterial **(B)** Bray–Curtis indices of the rhizosphere samples of eight cotton cultivars grown in the soils collected from the three sites: four cultivars from *Gossypium barbadense* (GB) and the other four from *Gossypium hirsutum* (GH).

For fungi, there were clear separations of samples along the three soil types although there was considerable scattering of samples within each soil type, especially for soils from Alaer and Shihezi (Figure 4A). Chytridiomycota and Rozellomycota (in the opposite directions) were the two important classes separating samples along the first NMDS dimension. Along the second NMDS dimension, sample separation was primarily due to the “Unknown” classes. ADONIS permutational analysis confirmed the importance of the soil type in affecting fungal rhizosphere communities, accounting for 43.4% of the variability in the Bray–Curtis indices. About 49.1% of the variability in the Bray–Curtis indices remained unexplained. Two cotton species did not differ significantly, but the differences between cultivars within species were close to statistical significance at 5% (*P* = 0.054), accounting for 6.4% of the total variability.

Similarly for the bacterial communities, there were clear separations of samples along the three soil types, but with less scattering among samples within each soil type than the fungal communities (Figure 4B). Samples for plants grown in the soils from Shihezi and Alaer were separated into two subgroups, not apparently related to cultivars (Figure 4B). Tenericutes and Chlamydiae (in the opposite directions) were the two important classes differentiating samples along the first NMDS dimension. Along the second NMDS dimension, Fusobacteria, and Gemmatimonadetes, as opposed to Spirochaetes and Ignavibacteriae, were important in separating samples. The differences among the three soil types accounted for 78.5% of the variability in the bacterial Bray–Curtis indices; 18.2% of the variability remained unexplained. Neither the two species nor cultivars within species differed significantly.

### Differential abundance analysis

Of 5,768 fungal OTUs, only 1,125 OTUs passed the default DESeq2 filters for differential abundance analysis between the two cotton species. Twenty-one OTUs differed in their differential relative abundance between the two species, including several mushroom taxa groups. Only six of the 21 OTUs can be confidently assigned to the species rank; seven of the 21 OTUs cannot be confidently assigned to the phylum rank. In 13 of the 21 OTUs, *G. barbadense* had a higher relative abundance than *G. hirsutum* (Table 3) with an average log_2_FoldChange (LFC) of 4.59, including all six OTUs with the highest sequence reads number among the 21 OTUs. For the other eight OTUs, *G. barbadense* had a lower relative abundance than *G. hirsutum*, with an average LFC of −5.61 (Table 3).

**TABLE 3:** Summary of differential abundance comparison of individual fungal operational taxonomic units (OTUs) of the rhizosphere between the two cotton species. Only 21 OTUs had significant differences [Benjamini-Hochberg (BH) adjusted *P* < 0.05] in the relative abundance between the two cotton species: positive log_2_FoldChange indicates that the relative abundance is higher in *Gossypium barbadense* than in *Gossypium hirsutum*.

OTU ID	Taxonomy	baseMean	log_2_FoldChange
OTU406	Agaricomycetes (c)	81	4.22
OTU111	Ascomycota (p)	255	–5.97
OTU100	*Aspergillus caespitosus*	292	–4.78
OTU77	Chytridiomycetes (c)	544	6.58
OTU87	*Coprinellus canistri*	715	5.74
OTU597	Fungi (k)	11	–6.79
OTU792	Fungi (k)	12	–6.18
OTU488	Fungi (k)	20	3.66
OTU467	Fungi (k)	24	4.25
OTU383	Fungi (k)	30	–5.97
OTU224	Fungi (k)	68	4.54
OTU1239	Fungi (k)	101	6.11
OTU560	*Limacella delicata*	11	–5.42
OTU677	*Minimedusa polyspora*	7	–6.19
OTU228	Paratritirachium (g)	60	4.46
OTU3642	Pleosporales (o)	18	3.61
OTU13	Pleosporales (o)	10,515	3.45
OTU43	Sebacinales (o)	1,464	4.28
OTU108	Sordariomycetes (c)	413	–3.66
OTU35	*Suillus placidus*	2,395	4.68
OTU51	*Tricholoma portentosum*	1,391	4.06

Of 4,941 bacterial OTUs, 2,731 OTUs passed the default DESeq2 filters and were compared for their relative abundance between the two species. The relative abundance differed between the two species for 72 OTUs, of which three OTUs had high reads numbers (> 4,300). Only six of the 72 OTUs can be confidently assigned to the species rank (Table 4); for many of the 72 OTUs, taxonomy assignment cannot be confidently made below the order level. In 47 of the 72 OTUs, *G. barbadense* had a higher relative abundance than *G. hirsutum* (Table 4), with an average LFC of 1.20; for the other 25 OTUs, *G. barbadense* had a lower relative abundance than *G. hirsutum*, with an average LFC of −1.18. Interestingly, for all 22 OTUs of Acidobacteria, *G. barbadense* had a higher relative abundance than *G. hirsutum* (Table 4).

**TABLE 4:** Summary of differential abundance comparison of individual bacterial operational taxonomic units (OTUs) of the rhizosphere between the two cotton species. Only 72 OTUs had significant differences [Benjamini-Hochberg (BH) adjusted *P* < 0.05] in the relative abundance between the two cotton species: positive log_2_FoldChange indicates that the relative abundance is higher in *Gossypium barbadense* than in *Gossypium hirsutum*.

OTU_ID	Rank	baseMean	log_2_FoldChange
OTU80	Acidimicrobiales (o)	444.83	–0.94
OTU1976	Actinobacteria (c)	23.90	–2.08
OTU398	Actinobacteria (c)	112.73	–1.19
OTU1147	Actinobacteria (c)	84.03	–0.67
OTU1798	Actinobacteria (c)	941.13	0.36
OTU1885	Actinomycetales (o)	35.00	–1.17
OTU2300	Actinomycetales (o)	81.33	–0.97
OTU1468	Actinomycetales (o)	63.55	–0.85
OTU6	Actinomycetales (o)	4,976.47	0.38
OTU168	*Algoriphagus* (g)	358.08	–0.54
OTU519	Anaerolineaceae (f)	151.35	0.76
OTU1032	Anaerolineaceae (f)	95.07	1.05
OTU992	*Aquicella siphonis*	90.46	0.79
OTU1231	*Azoarcus* (g)	65.10	–1.34
OTU1933	Bacteria (k)	17.14	–1.75
OTU322	Bacteria (k)	184.56	–1.25
OTU391	Bacteria (k)	112.62	–0.86
OTU14	Bacteria (k)	4,461.55	–0.63
OTU494	Bacteria (k)	441.24	0.44
OTU162	Bacteria (k)	406.21	0.52
OTU1344	Bacteria (k)	198.13	0.63
OTU87	Bacteria (k)	817.70	0.64
OTU281	Bacteria (k)	361.09	0.64
OTU758	Bacteria (k)	111.63	0.74
OTU146	Bacteria (k)	375.13	0.81
OTU1078	Bacteria (k)	75.85	0.82
OTU739	Bacteria (k)	63.77	1.03
OTU1712	Bacteria (k)	36.83	1.23
OTU1565	Bacteria (k)	46.15	2.12
OTU704	*Bacteroides fragilis*	143.58	6.81
OTU959	Chloroplast (f)	62.97	1.00
OTU4260	*Conexibacter arvalis*	25.17	–1.28
OTU912	*Cupriavidus* (g)	59.49	–2.31
OTU1720	Flavobacteriaceae (f)	81.78	–1.50
OTU2875	Flavobacteriaceae (f)	38.93	2.99
OTU2771	*Fusobacterium* (g)	26.54	4.68
OTU341	Acidobacteria Gp16 (g)	617.65	0.50
OTU2076	Acidobacteria Gp16 (g)	405.17	0.75
OTU188	Acidobacteria Gp16 (g)	282.19	0.90
OTU777	Acidobacteria Gp21 (g)	173.58	0.61
OTU583	Acidobacteria Gp25 (g)	194.12	0.85
OTU1529	Acidobacteria Gp25 (g)	59.16	1.14
OTU445	Acidobacteria Gp4 (g)	939.81	0.61
OTU90	Acidobacteria Gp4 (g)	1,083.86	0.68
OTU813	Acidobacteria Gp4 (g)	480.83	0.69
OTU17	Acidobacteria Gp4 (g)	4,302.01	0.72
OTU980	Acidobacteria Gp6 (g)	841.13	0.51
OTU745	Acidobacteria Gp6 (g)	293.80	0.57
OTU1129	Acidobacteria Gp6 (g)	276.60	0.65
OTU1234	Acidobacteria Gp6 (g)	214.80	0.82
OTU2281	Acidobacteria Gp6 (g)	122.88	0.84
OTU3157	Acidobacteria Gp6 (g)	62.44	0.84
OTU2695	Acidobacteria Gp6 (g)	56.69	0.96
OTU560	Acidobacteria Gp6 (g)	128.58	0.97
OTU989	Acidobacteria Gp6 (g)	84.91	1.01
OTU963	Acidobacteria Gp6 (g)	68.65	1.04
OTU648	Acidobacteria Gp7 (g)	291.35	0.53
OTU985	Acidobacteria Gp9 (g)	269.36	1.07
OTU153	*Haloferula* (g)	241.46	0.78
OTU503	*Hydrogenophaga* (g)	328.25	–1.11
OTU473	*Marinobacterium litorale*	154.45	–0.99
OTU2282	Myxococcales (o)	17.84	–1.56
OTU4751	Myxococcales (o)	23.36	2.11
OTU2479	Opitutaceae (f)	19.01	1.60
OTU1974	*Panacagrimonas perspica*	55.64	–0.88
OTU1043	*Ruminococcus faecis*	124.80	4.69
OTU1524	Saccharibacteria_genera _incertae_sedis (g)	29.05	–1.84
OTU414	*Steroidobacter agariperforans*	1,271.46	–0.40
OTU1148	Subdivision3_genera _incertae_sedis (g)	48.96	–0.86
OTU328	Subdivision3_genera _incertae_sedis (g)	149.01	–0.84
OTU2531	*Thiohalomonas denitrificans*	32.45	–1.62
OTU585	Verrucomicrobiaceae (f)	121.21	1.37

## Discussion

The two cotton species (eight genotypes) used in the current study varied greatly in their resistance to *V. dahliae*, but no major difference was observed in the rhizosphere microbial community structure of the three soil types. Similar findings for microbial community compositions were observed in the rhizosphere of wheat cultivars (Li et al., 2020). Interestingly, the findings of the current study contradict those of our previous work which revealed that rhizosphere and endosphere microbial communities differed significantly among cotton genotypes with varying degrees of wilt resistance (Wei et al., 2019). This noticeable difference in the microbial communities could be attributed to the physicochemical properties of soils used in this study. This study was performed with three types of soil (obtained from 1,000 to 3,000 km apart) having variable physicochemical properties, whereas the prior study was conducted on the same soil (from Anyang) with different cotton genotypes. As plants were grown under the same conditions in the bulk soil collected from various cities (distance greater than 1,000 km) harboring diverse and variable microbiome, there is a need to consider both the microbial reservoirs and root exudates when interpreting the effects of plant genotypes on the rhizosphere.

Soil is a reservoir of diverse microbial communities with a range of functions that cause a significant impact on soil health. Based on the findings of this study, it was concluded that: (1) soil is a key factor, acting as a reservoir and determining rhizosphere microbial community structures, and (2) plant genotype can play a certain role in shaping their rhizosphere microbial communities, especially bacterial communities. Previous literature demonstrated that the abundance and diversity of microbial communities can be affected by soil type, plant genotype, root exudates, and various environmental factors. Among them, soil type with a range of factors such as structure, soil texture, moisture contents, pH, salinity, and fertility primarily impacts the soil microbial communities (Bach et al., 2010; O’Brien et al., 2019). In this study, Proteobacteria, Actinobacteria, Acidobacteria, Firmicutes, and Bacteroidetes were the dominant phyla (> 5% relative abundance) in the three soil types grown with eight genotypes. It was noted that these bacterial phyla were either promoted or inhibited to different degrees, but no significant difference was observed in the abundance of these bacterial phyla, particularly between soil types; however, the total reads for the composition of these phyla fluctuated between the rhizosphere soils. The alpha diversity of rhizosphere bacteria was diverse in the soil from Anyang, but significantly promoted in the other two soil types. In addition, the beta diversity between cultivars and species did not differ significantly. These results of this study presumably occurred because of the following three reasons: (i) difference in soil indigenous microbial community structure, (ii) difference in soil physical and chemical properties, and (iii) the effects of cotton genotypes. Therefore, we speculate that the physical and chemical characteristics of soil maximize the reproduction of bacteria because soil conditions mainly regulate the overall soil microbial community with specific members colonizing plant rhizosphere, rhizoplane, and endosphere, which is essentially the concept of soil as a microbial seed bank (Zarraonaindia et al., 2015).

On the contrary, root exudates can attract indigenous microorganisms from the nearby soil environment and promote their abundance, thus responsible for the variation in the abundance and diversity of rhizosphere microbial community, which is evident from previous literature (Lundberg et al., 2012; Peiffer et al., 2013; Bonito et al., 2014; Mendes et al., 2018; Veach et al., 2019). Therefore, the microorganisms that were originally present in soil failed to adapt to the changing environment, experienced a reduction in their abundance, or even disappeared. Inversely, a portion of microbes was promoted and multiplied greatly, thus creating a competitive relationship with other microbial species or secreting antimicrobial substances to inhibit the colonization of other bacterial species. Therefore, the richness of rhizosphere bacteria in the three soil types was significantly different.

Additionally, compared with Shihezi and Alar, the rhizosphere bacterial population of genotypes growing in the soil of Anyang was more diverse and had a greater proportion of Proteobacteria, Actinobacteria, and Firmicutes. Proteobacteria are dominant phyla in soil that are involved in N-cycle by oxidizing ammonia into nitrite, global carbon cycle, and the absorption of iron, nitrogen, and sulfur elements, which is consistent with the fact that Proteobacteria grow rapidly and can quickly utilize carbon source substances secreted by rhizosphere (Spain et al., 2009; Shin et al., 2015). Gram-positive bacteria, such as Actinobacteria and Firmicutes, are reported to be functional phyla that can inhibit the growth of pathogens and participate in the carbon cycle of the earth (Lee et al., 2021; Ling et al., 2022). Therefore, rhizosphere microorganisms of cotton varieties with different resistances play an important role in soil disease control. Moreover, 22 Acidobacteria taxa groups have significantly high relative abundance in *G. barbadense* compared with *G. hirsutum* in this study. This is attributed to different types of cotton cultivars (*G. hirsutum* and *G. barbadense*), because previously it was suggested that neither *Bt* cotton nor conventional cotton cultivation had any significant effect on the total abundance of soil bacteria (Lv et al., 2022). But, in the *Bt* cotton, the abundance of Acidobacteria decreased by 1.87% compared with its control group, and in the conventional cotton cultivars, the abundance of Acidobacteria was higher by 1.66% than its control group (Lv et al., 2022). Therefore, in this study it is proven that *Bt* cotton cultivation could affect the soil bacterial community structure and functions. Consequently, the difference in the abundance of Acidobacteria in different types of cotton was owing to the difference in soil–microbe interactions. Moreover, Acidobacteria are one of the most dominant phyla in diverse soil habitats, representing 5–50% of the total bacterial community (Lee et al., 2008; Foesel et al., 2014; Dedysh and Damsté, 2018), and their genome possesses a comprehensive physiological set of genes that allow them to adapt to various ecological niches, enabling them to participate in carbon usage, nitrogen assimilation, metabolism of iron, Antimicrobial activities, abundance of transporters, oxygen and hydrogen utilization, stress and starvation response, and secondary metabolite biosynthesis (Kielak et al., 2009; Eichorst et al., 2018; Kalam et al., 2020). Acidobacteria also contribute to promoting plant growth and protecting against phytopathogens by producing phytohormone indole-3-acetic acid (IAA), siderophores, as well as carotenoids (Wang et al., 2014; Kielak et al., 2016; Pinto et al., 2021). Several studies showed that Acidobacteria act as slow-acting decomposers of plant-, fungi-, and insect-derived polymers (Dedysh and Yilmaz, 2018). However, further study is needed to determine whether the higher abundance of Acidobacteria in the rhizosphere of *G. barbadense* contributes to its resistance against Verticillium wilt or not.

## Conclusion

This study validates the contribution of soil origin and plant genotype in driving rhizosphere microbiome assembly. Specifically, soil origin appears to result in shifts of key bacterial and fungal groups in rhizosphere within differing plant genotypes. Initiatives using cotton in a fiber production may need to consider not only genotype but also belowground microbiome that is recognized as the second genome of plants. These results should be a key consideration for future plant–soil–microbial interactions research attempting to integrate plant growth and microbiomes.

## Data availability statement

The datasets presented in this study can be found in online repositories. The names of the repository/repositories and accession number(s) can be found below: https://www.ncbi.nlm.nih.gov/, PRJNA836023.

## Author contributions

FW, XX, and HZ planned and designed the research and experiments. CY, HY, ZM, ZF, HF, LZ, and YZ performed the experiments. FW, XX, and GD analyzed the data. FW, CY, and XX wrote the manuscript. FW acquired the funds for the study. All authors read and approved the final manuscript.

## Footnotes

1
http://cutadapt.readthedocs.io/en/stable/

2
https://github.com/torognes/vsearch/

3
http://www.drive5.com/usearch/manual/uchime_algo.html

4
https://www.drive5.com/usearch/manual/sintax_algo.html
