# Supplementary material for: Influence of plant genotype and soil on the cotton rhizosphere microbiome
Source: Front Microbiol. 2022 Sep 20;13:1021064. doi: 10.3389/fmicb.2022.1021064 (PMC9530387; doi:10.3389/fmicb.2022.1021064)
Supplement: Supplementary file 1 [file Data_Sheet_1.docx]

**Table S1.** Verticillium wilt summary of those eight cultivars included in the study; wilt inoculum CFU values and disease indices in the field experiment were the averages over three replicates plots; wilt disease indices of the greenhouse inoculation trials in sterilized soils (inoculated *Verticillium dahliae*) were summarized over three replicates experiments.

| Cotton species | Cultivar | Disease incidence (%) | Disease index |
| --- | --- | --- | --- |
| *Gossypium hirsutum* | TM-1 | 95.82 | 49.07 |
| *G. hirsutum* | Zhongmiansuo24 | 87.38 | 38.06 |
| *G. hirsutum* | Zhongmiansuo35 | 76.79 | 33.98 |
| *G. hirsutum* | Lumianyan21 | 76.30 | 28.05 |
| *G. barbadense* | Hai7124 | 3.15 | 0.79 |
| *G. barbadens* | Xinhai21 | 2.83 | 0.71 |
| *G. barbadens* | Xinhai25 | 5.06 | 1.27 |
| *G. barbadens* | 3-79 | 5.87 | 4.40 |


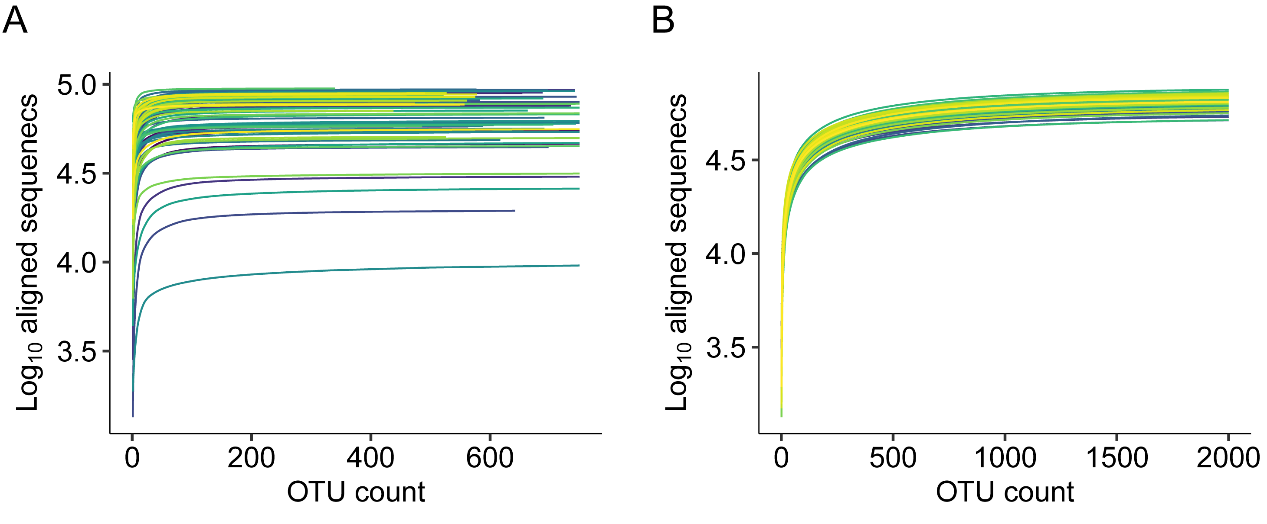


**Figure S1.** Rarefication curves of fungal sequences for a selected number of samples, including the sample with the least number of reads included in all OTUs.


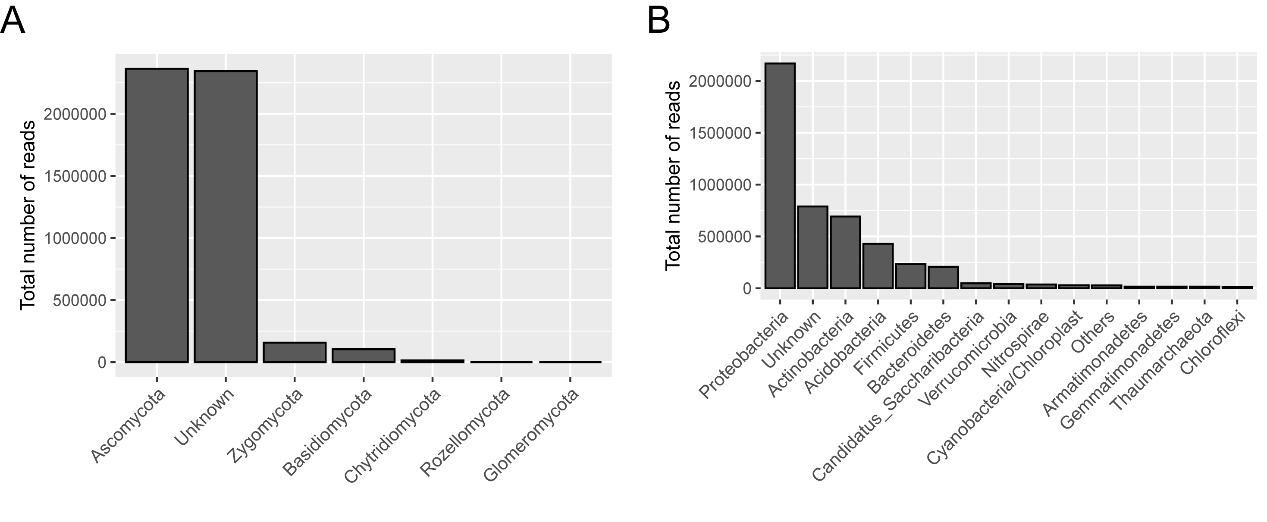


**Figure S2.** Number of sequences reads in each phylum; when sequences (OTUs) cannot be assigned to a phylum at the confidence level of 80%, they were assigned to the “Unknown” group.


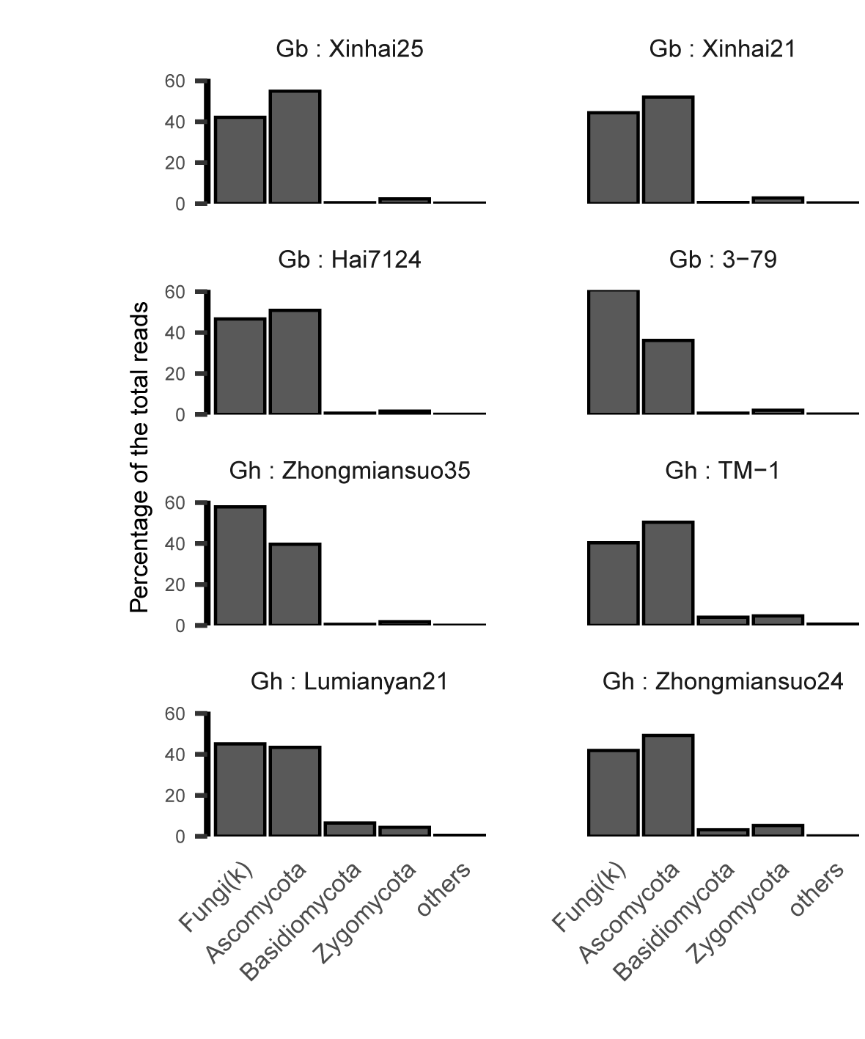


**Figure S3.** Percentage of fungal sequence reads in each Phylum within each cotton cultivar studied.


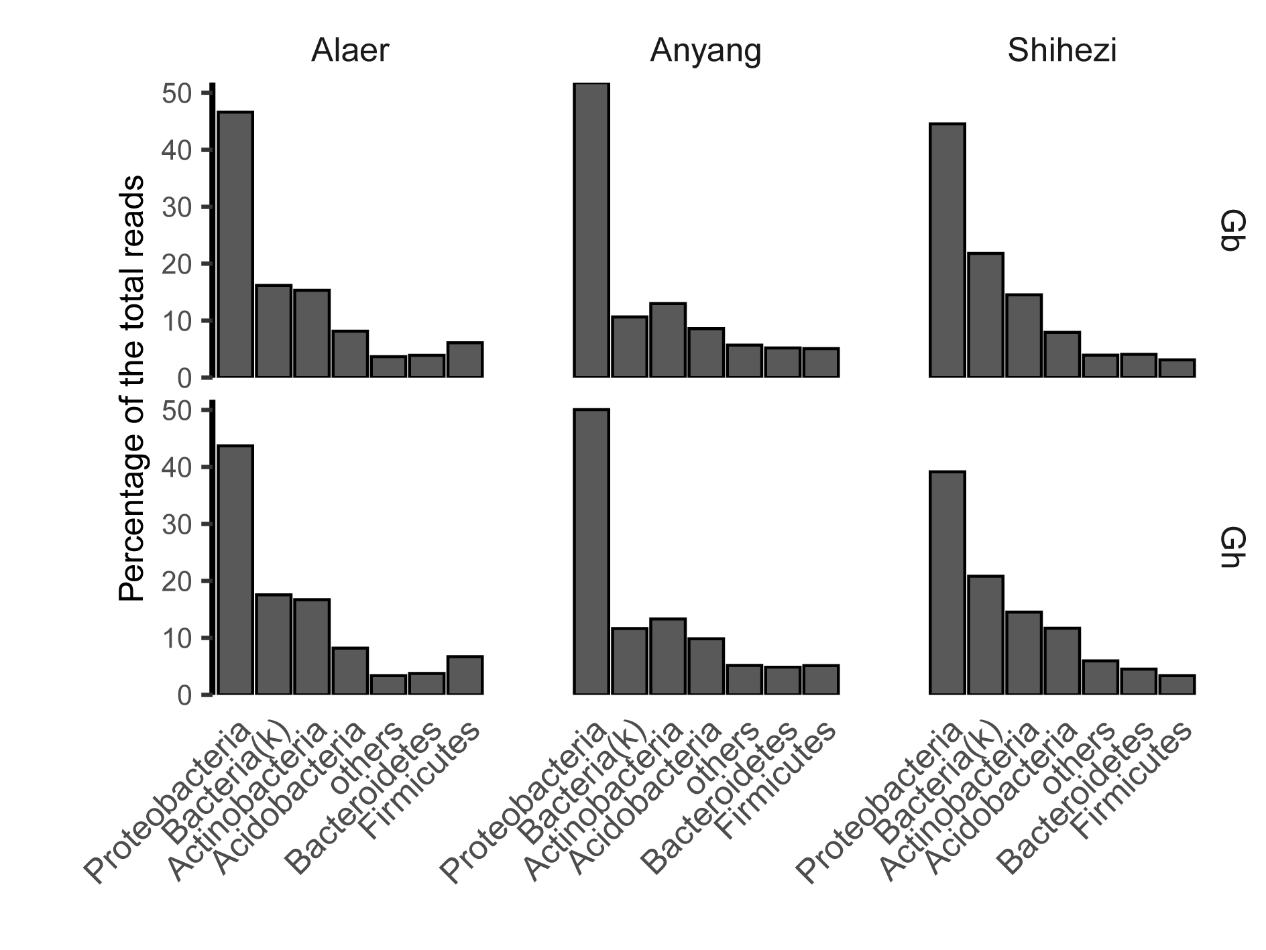


**Figure S4.** Percentage of bacterial sequence reads in each Phylum within combination f cotton species and soil type.
